# Constrictive Pericarditis with Extensive Calcification and Caseous Necrosis

**DOI:** 10.21470/1678-9741-2019-0224

**Published:** 2020

**Authors:** Marco Antônio Volpe, Jorge Edwin Morocho Paredes, Emerson Maron, Isaac Samuel Moscoso Sanchez, João Alberto Pastor de Oliveira, Luiza Zita D’Albuquerque Silveira

**Affiliations:** 1 Centro para Assistência Integral em Cardiologia (CERAIC), São Paulo, SP, Brazil.; 2 Hospital IGESP, São Paulo, SP, Brazil.

**Keywords:** Pericarditis, Constrictive, Pericardiectomy, Ventricular Function, Right, Pericardium, Heart Failure, Treatment Outcome

## Abstract

Constrictive pericarditis is a disease where loss of pericardial elasticity and restriction of filling of the cardiac chambers occurs. It is most often seen as an associated symptom of heart failure. Pericardiectomy provides effective treatment for patients with symptomatic constrictive pericarditis, although high rates of morbidity and mortality are related to the procedure. We present a case with extensive calcification, massive caseous necrosis and an important impairment of right ventricular function successfully operated in our institution.

**Table:** 

Abbreviations, acronyms & symbols	
HIV	= human immunodeficiency virus

## INTRODUCTION

Constrictive pericarditis is an uncommon disease with multiple causes. Defined as a cicatricial process leading to thickening, hardening and, in most cases, calcification of the pericardium which ultimately result in loss of pericardial elasticity and restriction of filling of the cardiac chambers^[1,2]^. During disease progression, patients develop signs and symptoms of congestion as a result of restriction to ventricular filling^[1,2]^. Pericardiectomy remains the only effective treatment for patients with symptomatic constrictive pericarditis although high rates of morbidity and mortality are still reported for this procedure^[3-5]^. Only a few publications have identified organ dysfunction as independent risk factors for mortality^[3,4]^. The aim of this report was to demonstrate the good results of the surgical treatment for constrictive pericarditis despite extensive calcification, massive caseous necrosis and an important impairment of right ventricular function.

## CASE REPORT

GSA, male, 55 years old, white, forklift operator, natural and from the state of São Paulo, was referred to the office reporting dyspnea on efforts with progressive worsening in the last two months, asthenia, inappetence and weight loss. He reported periods of night sweats but denied a fever. He referred to hypothyroidism and dyslipidemia on treatment with levothyroxine sodium and simvastatin. Physical examination was afebrile, eupneic and with mild jugular stasis. In the chest examination no adventitious noises were observed in the pulmonary auscultation and, in cardiac auscultation, the rhythm was irregular, without murmurs and the sounds were normal. No paradoxical pulse was observed. In the examination of the abdomen there was discrete hepatomegaly without other changes. Biochemical tests were normal, serology for human immunodeficiency virus (HIV) was negative, and the search for alcohol-acid-resistant bacilli in the sputum was negative. On the electrocardiogram, atrial fibrillation was evidenced. In the simple chest radiographs, there was extensive pericardial calcification involving right ventricular chamber and right atrial chamber (Figure 1A). In the color doppler echocardiogram it was observed the presence of echogenic image prior to the right atrium and ventricle, mainly restricting the mobility and contractility of the basal mean portion of the ventricular chamber; asynchronous movement of the whole right ventricle and interventricular septum compromising the systolic-diastolic function of this chamber; dilated hepatic veins and inferior vena cava with variation of expandability reduced to the maneuvers and impairment of left ventricular systolic function. Magnetic resonance of the thorax revealed signs of pericardial thickening with surrounding calcifications, with the largest thickness, about 3 cm, in the anterior and lower anterior pericardial walls (Figure 1B). The hemodynamic study showed significant diffuse pericardial calcification and absence of obstructive coronariopathy. With these findings, pericardiectomy was indicated. Access to the thoracic cavity was by median sternotomy, after which a distended pericardium was seen in the lower third. The opening immediately exposed a large volume of a doughy material of dark brown color and necrotic appearance (caseous necrosis). A great amount of this collection was promptly removed from below the diaphragmatic wall of the right ventricle where it had been exerting a great compressive effect (Figure 2A). During surgery, a large volume of calcium was found in the pericardium, most of which strongly adhered to the right chambers, compressing the right ventricle, particularly. Careful removal of the thickened pericardium and calcium was required, and in view of the hemodynamic instability, the risk of cardiac perforation and the posterior extension of pericarditis, the use of extracorporeal circulation was chosen. Extracorporeal circulation was then established by drainage of both vena cava and arterial infusion through the ascending aorta. Under total aortic clamping and cardioplegic arrest, with Custodiol®, excision of the thickened pericardium and calcium were performed, which extended posteriorly until the pulmonary veins’ debouchment. After complete removal of pericardium, calcium, and caseous necrosis, the heart was fully released (Figure 2B). Extracorporeal circulation was then discontinued, with the help of inotropic drugs, as soon as the hemodynamic conditions allowed. The surgery was completed with revision of hemostasis, mediastinal and retrocardiac drainage and finally closure of the thorax. Patient remained under intensive care unit for 5 days, a longer period than usual, necessary for weaning vasoactive drugs and readaptation to the new hemodynamic picture. An anatomopathological study revealed chronic non-specific pericarditis with diffuse hyalinized fibrosis, vascular neoformation and extensive dystrophic calcification. The culture was negative for the presence of alcohol-acid-resistant bacilli. The patient presented a good evolution and was discharged from hospital to resume his daily life. The Doppler echocardiogram before hospital discharge revealed a significant improvement with biventricular normofunction. Despite the fact that the tests did not detect tuberculosis, due to the strongly suggestive clinical picture, antituberculosis (Rifampicin, Isoniazid, Pirazinamide) was started in the first phase and (Rifampicin, Isoniazid) in the second phase.

**Fig. 1A:**
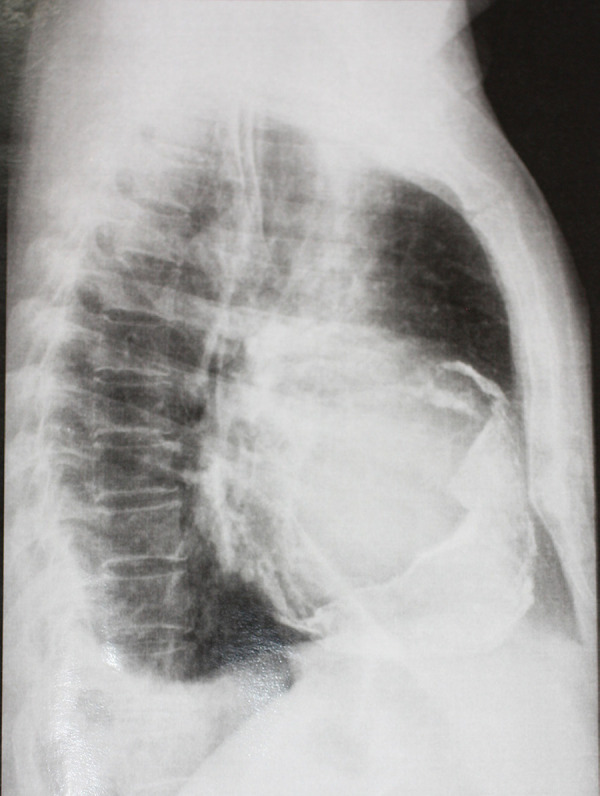
Chest X-ray.

**Fig. 1B:**
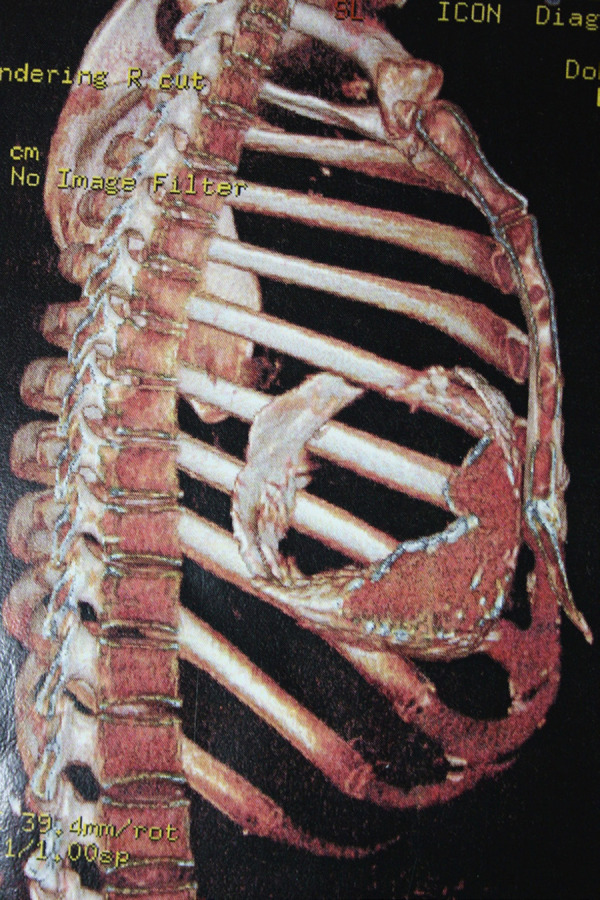
Magnetic resonance imaging of the chest.

**Fig. 2A:**
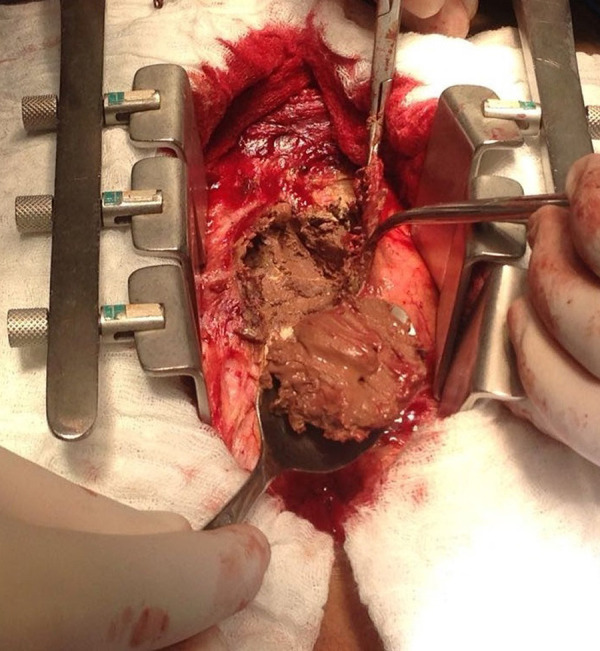
Removal of necrotic material which was compressing right ventricle.

**Fig. 2B:**
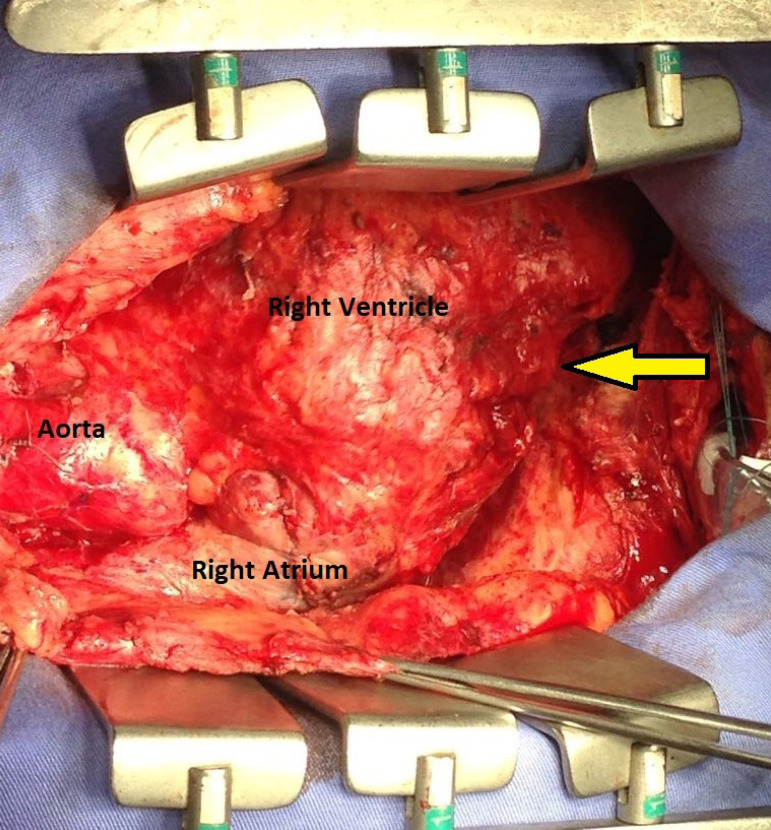
Heart released showing the extrinsic compression marks in the right ventricle (yellow arrow).

## DISCUSSION

Constrictive pericarditis is a less common disease and often has a late diagnosis^[1]^. All cardiac chambers can be affected with constriction resulting in restriction to diastolic filling^[1,2]^. The causes are diverse, but in developing countries the main etiology is tuberculosis^[1,6]^. It is most often manifested as right heart failure^[1]^ , fact also observed in the present case. Due to ventricular filling restriction, ventricular diastolic pressures increase, leading to large mean atrial pressures^[1,2]^, which was also seen in the aforementioned patient and what alerted the medical team to the need of careful dissection of the firm adherences to the cardiac chambers, especially for those performed before cardiopulmonary bypass.

The imaging exams - chest radiographs, echocardiogram, hemodynamic study and magnetic resonance of the thorax - are fundamental in confirming the diagnosis and surgical programming. They allow the surgical team to analyze the thickness of the pericardium and myocardium, the filling pressures of the chambers, the presence of effusion and the signs as well as the degree of calcification^[1]^. Magnetic resonance imaging is of great value for the differential diagnosis between constrictive and restrictive pericarditis^[1]^. In the present case, the images of the radiographs and magnetic resonance of the thorax were decisive to clarify the extent of calcification and the large volume of associated necrotic material leading to the important cardiac compression, particularly of the right ventricle.

What was seen during the surgery confirmed it was an extensive constrictive pericarditis with major calcification associated with a large volume of necrotic (caseous) material. In a relevant study with 20 years of experience, Vistarini et al.^[3]^ report a 9% in hospital mortality for pericardiectomy and that surgery performed within 6 months after the onset of symptoms is associated with lower mortality. According to the authors, this agility in performing the surgical procedure prevents clinical deterioration. In this context they refer to hepatomegaly as a worse prognostic factor since it is directly linked to the evolution of the disease as it reflects an advanced stage of heart failure resulting from persistent pericarditis. These findings were corroborated by us and seem quite reasonable once such surgeries are extensive, requiring important tissue dissection and long anesthetic period, which are crucial factors for borderline patients. These authors also showed a trend towards a worse outcome, but without statistical significance, for variables such as age, advanced functional class and pericardial calcification. This fact was also confirmed in the patient in question. Thus, although pericardiectomy remains associated with high operative mortality^[3,4]^, the long-term outcome is favorable and surgical treatment is effective in improving functional class in most patients leading to the disappearance of the symptoms of heart failure as in the case here presented.

**Table:** 

Author's roles & responsibilities	
MAV	Substantial contributions to the conception or design of the work; or the acquisition, analysis, or interpretation of data for the work; drafting the work or revising it critically for important intellectual content; final approval of the version to be published
JEMP	Substantial contributions to the conception or design of the work; or the acquisition, analysis, or interpretation of data for the work; drafting the work or revising it critically for important intellectual content; final approval of the version to be published
EM	Substantial contributions to the conception or design of the work; or the acquisition, analysis, or interpretation of data for the work; drafting the work or revising it critically for important intellectual content; final approval of the version to be published
ISMS	Substantial contributions to the conception or design of the work; or the acquisition, analysis, or interpretation of data for the work; drafting the work or revising it critically for important intellectual content; final approval of the version to be published
JAPO	Substantial contributions to the conception or design of the work; or the acquisition, analysis, or interpretation of data for the work; drafting the work or revising it critically for important intellectual content; final approval of the version to be published
LZDAS	Substantial contributions to the conception or design of the work; or the acquisition, analysis, or interpretation of data for the work; drafting the work or revising it critically for important intellectual content; final approval of the version to be published
